# Quantification of histone H2AX phosphorylation in white blood cells induced by *ex vivo* gamma irradiation of whole blood by both flow cytometry and foci counting as a dose estimation in rapid triage

**DOI:** 10.1371/journal.pone.0265643

**Published:** 2022-03-23

**Authors:** Rujira Wanotayan, Sarinya Wongsanit, Kanokporn Boonsirichai, Kasama Sukapirom, Sakchai Buppaungkul, Putthiporn Charoenphun, Pucharee Songprakhon, Kulachart Jangpatarapongsa, Pimpon Uttayarat

**Affiliations:** 1 Faculty of Medical Technology, Department of Radiological Technology, Mahidol University, Nakhon Pathom, Thailand; 2 Nuclear Technology Research and Development Center, Thailand Institute of Nuclear Technology (Public Organization), Ongkarak, Nakhon Nayok, Thailand; 3 Faculty of Medicine Siriraj Hospital, Siriraj Center of Research Excellence in Microparticle and Exosome in Diseases, Research Department, Bangkok, Thailand; 4 Secondary Standard Dosimetry Laboratory (SSDL), Bureau of Radiation and Medical Devices, Ministry of Public Health, Bangkok, Thailand; 5 Faculty of Medicine Ramathibodi Hospital, Division of Nuclear Medicine, Department of Diagnostic and Therapeutic Radiology, Mahidol University, Nakhon Pathom, Thailand; 6 Division of Molecular Medicine, Faculty of Medicine Siriraj Hospital, Research Department, Mahidol University, Bangkok, Thailand; 7 Faculty of Medical Technology, Center for Research and Innovation, Mahidol University, Nakhon Pathom, Thailand; Central Research Institute of Electric Power Industry (CRIEPI), JAPAN

## Abstract

A quick, reliable, and reproducible biological assay to distinguish individuals with possible life-threatening risk following radiological or nuclear incidents remains a quest in biodosimetry. In this paper, we examined the use of a γ-H2AX assay as an early dose estimation for rapid triage based on both flow cytometry and image analyses. In the experiment, whole blood from 11 donors was irradiated *ex vivo* inside a water phantom by gamma rays from Co-60 at 0.51 Gy/min. After the lysis of red blood cells, the white blood cells were collected for immunofluorescence labeling of γ-H2AX, CD45, and nuclear stained for signal collection and visualization. Analysis by flow cytometry showed that the relative γ-H2AX intensities of lymphocytes and granulocytes increased linearly with absorbed doses from 0 to 6 Gy with a large variation among individuals observed above 2 Gy. The relative γ-H2AX intensities of lymphocytes assessed by two different laboratories were highly correlated (ICC = 0.979). Using confocal microscopic images, γ-H2AX foci were observed to be discretely distributed inside the nuclei and to increase proportionally with doses from 0 to 2 Gy, whereas large plagues of merged foci appeared at 4 and 6 Gy, resulting in the saturation of foci counts above 4 Gy. The number of total foci per cell as well as the number of foci per plane were significantly different at 0 vs 1 and 2 vs 4 Gy doses (*p* < 0.01). Blind tests at 0.5 Gy and 1 Gy doses showed that dose estimation by flow cytometry had a mean absolute difference of less than 0.5 Gy from the actual value. In conclusion, while flow cytometry can provide a dose estimation with an uncertainty of 0.5 Gy at doses ≤ 1 Gy, foci counting can identify merged foci that are prominent at doses ≥ 4 Gy.

## Introduction

A major challenge in biodosimetry is the rapid reconstruction of absorbed dose to prioritize proper treatment for possibly exposed individuals [1–3]. In the absence of physical dosimetry on the eve of unforeseeable radiological or nuclear incidents such as Chernobyl and Fukushima [4], a high throughput biodosimetry technique is required to triage large-scale populations. Over the past decades, several biodosimetric assays have been developed for estimating the absorbed dose as well as evaluating the extent of biological damage [3]. Among these methods, the dicentric chromosome assay (DCA) remains the gold standard due to its specificity, high sensitivity, capability to identify partial-body exposure to ionizing radiation (IR), and also ability to perform dose estimation in retrospect up to about 3–6 months after an incident [3]. Despite the use of automation and network in scoring dicentric chromosomes, however, the time required to prepare non-reproducible cultures of lymphocytes as well as the need for skilled cytogeneticists pose major limitations to its use in a large-scale radiation emergency [4–8]. Therefore, there is still a need to develop a quick and high throughput assay to handle a large number of samples [4].

Protein-based biomarkers, such as phosphorylated H2AX histone [9] and the p53 binding protein 53BP1 [10], provide attractive alternatives to the cytogenetic-based assay due to their strong correlation with DNA double-strand breaks (DSBs), high throughput, automation capacity, reliability, and reproducibility [5,11–13]. At the early stage of cellular response to IR-induced DSBs, phosphorylation of hundreds to thousands of H2AX proteins at the 139^th^ serine residue, or γ-H2AX [14], occurs at damage sites which then recruit other signaling factors such as MDC1 and 53BP1 [11,12] to join the cascade of DNA repair. The presence of these γ-H2AX foci inside the cell nuclei can be visualized by immunofluorescence labeling and quantitated by intensity-based measurements such as flow cytometry and foci counting of microscopy images, which allowed for the quantitation of DNA DSBs that typically showed the linear response to IR doses [4]. Because γ-H2AX and 53BP1 are usually colocalized [6,11,13,15], much research has extensively utilized the γ-H2AX assays in various models including blood [1,6,12,13,16–22], buccal cells [23], cell lines [24,25], *in vivo* animal models [24,26], *ex vivo* skin [27], and skin equivalents [12]. Among these reports, the IR-induced γ-H2AX repair kinetics has been shown to be dependent of age [22,28,29], but not gender and smoking habit [12,18].

Variation in γ-H2AX expression among individuals at the same absorbed dose observed by flow cytometry analysis [16,20] and the kinetic disappearance of γ-H2AX expression from its peak to baseline within 24 h observed by both flow cytometry and foci counting [1,4,6,18,22] have been previously reported. Although these issues might post major limitations of γ-H2AX assay as a ‘dosimeter’ for the accurate dose estimation, the γ-H2AX assay is nevertheless still useful as an ‘indicator’ of IR exposure in triage [4,20]. Based on foci counting, previous studies by Redon *et al*. [12] and Horn *et al*. [13] reported the persistence of residual foci above baseline in lymphocytes 48 h and 96 h after receiving 2 and 4 Gy doses, respectively. Recently, more advanced high-throughput techniques such as the Rapid Automated Biodosimetry Tool (RABiT) [22,30,31], imaging flow cytometry platform [5,32], automated slide scanning [33] and ELISA assay [34] have been developed to facilitate γ-H2AX quantitation. Aside from biodosimetry, the γ-H2AX assay can also be applied to monitor the biological effects of patients and health workers that are exposed to radiation during diagnosis and therapy as well as workers in radiation facilities [4].

In this study, we focused on use of the γ-H2AX assay as a quick estimate to determine an absorbed dose based on the analyses by flow cytometry and foci counting of microscopy images. As part of Thailand’s biodosimetry network, our work represents the first attempt to establish a dose response curve based on the γ-H2AX biomarker as a complement to the already established calibration curves based on DCA and PCC assays [35]. Whole blood samples were irradiated by gamma rays and the dose response curves were constructed based on the relative γ-H2AX intensities and foci counts of lymphocytes, respectively, after which the accuracy of dose estimation was performed by blind tests. In the calculation of relative γ-H2AX intensities, our study shed a new light into the use of average γ-H2AX intensity at 0 Gy in the case of emergency when the baseline of exposed individual was not available. In addition, a preliminary exercise was performed on the use of flow cytometry for intercomparison study. In terms of dose response based on foci counts, the relationship between foci counts obtained from all focal planes and a single focal plane containing the maximum foci was established so that the latter can be used as an alternative in dose estimation.

## Materials and method

### Dose calibration with water phantom

**Fig 1:**
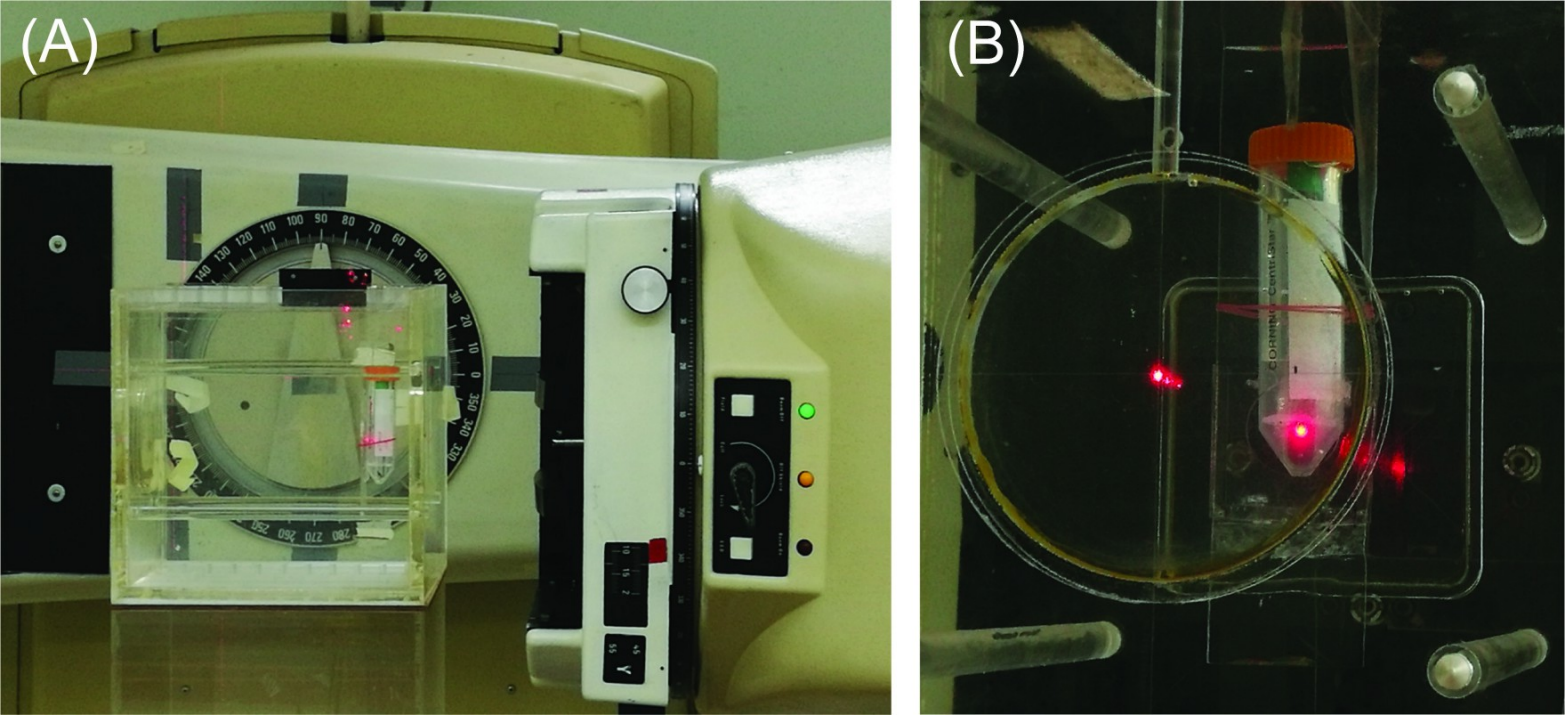
Setup of whole blood irradiation. (A) A water phantom containing blood sample was positioned at 60 cm in front of a Co-60 teletherapy machine with two orthogonal laser beams to aid the alignment. (B) Blood sample in a heparinized vacutainer tube was inserted into a 50-mL centrifuge tube containing a hollow ice cube during the irradiation process.

Irradiation of blood samples was conducted at the Secondary Standard Dosimetry Laboratory (SSDL), Bangkok, Thailand. A 30 cm x 30 cm x 30 cm water phantom was placed at a distance of 60 cm in front of a Co-60 teletherapy machine (Theratron Phoenix, Canada). Position of sample holder inside the water phantom was aligned using two orthogonal laser beams that projected onto the sidewalls of the water phantom (Fig 1A). Gantry and collimator parameters were set at 90 and 0 degrees, respectively, for an open field size of 10 cm x 10 cm. An ionization chamber connected to an electrometer was used for dose measurement inside the water phantom. The absorbed dose rate to water was determined according to IAEA guidelines (technical report series No. 398).

### Determination of absorbed dose by physical dosimeter

Optically stimulated luminescence (OSL) dosimeters, nanoDot^TM^ (Landuaer, USA), were chosen as a single point measurement of absorbed dose as they could be inserted inside blood collection tubes. The calibration curve of nanoDot^TM^ was performed at the Radiation Dose Measurement and Assessment Laboratory, Thailand Institute of Nuclear Technology (Public Organization) to determine the relative conversion factor (RCF) between delivered dose and sensitivity of nanoDot^TM^ dosimeter. The RCF was pre-generated by irradiating nanoDots^TM^ placed inside heparinized vacutainer tube (Becton Dickinson, USA) held in a water phantom with gamma rays and air kerma of 5 and 50 mGy following the ISO 4037–1:2019. The air kerma values were traceable to Physikalisch-Technishe Bundesanstalt, Germany.

In the experiment, four nanoDots^TM^ were placed inside the heparinized vacutainer tube held in the water phantom using the same setup shown in Fig 1 at SSDL. A total of five delivered exposures to gamma rays from Co-60 were made at 0.5, 1, 2, 4 and 6 Gy. Then, the reading of absorbed dose was assessed by a microSTAR reader at the Radiation Dose Measurement and Assessment Laboratory in which the beam quality and built-in software had been pre-calibrated with Cs-137 at 662 keV traceable to National Institute of Standards and Technology, USA. The energy response of nanoDots^TM^ to Cs-137 was 0.998, which was similar to that of Co-60, 0.995 according to the manufacturer.

### Blood collection and irradiation conditions

This project was approved by the Mahidol University Central Institutional Review Board (MU-CIRB 2019/119.1904). Informed consent was obtained from each volunteer prior to venipuncture. Peripheral blood samples were collected from 11 healthy volunteers (three males and eight females), aged 24–42 years without smoking habits, in heparinized vacutainer tubes (Becton Dickinson, USA) as aliquots of 1 mL per tube. Details of the experiments performed on the blood samples were summarized in S1 Table. Each blood aliquot in a heparinized vacutainer tube was inserted into a 50-mL centrifuge tube containing a hollow ice cube that served to cool the sample (Fig 1B). Samples were irradiated on ice to prevent DNA repair during exposure to IR [20]. The entire ensemble was then placed inside the water phantom and the samples were exposed to gamma rays at a dose rate of 0.51 Gy/min to obtain the total doses of 0.5–6 Gy. The blood sample was then incubated at 37°C for 45 min before being processed for immunofluorescence staining of γ-H2AX.

### Immunofluorescence staining

The staining protocol was adapted from Andrievski *et al*. [20]. Briefly, a 1-mL of the irradiated samples were fixed in 650 μL of 10% paraformaldehyde (Sigma, USA) for 30 min at room temperature, followed by a 30 min incubation in 0.12% (v/v) Triton X-100 diluted in phosphate buffered saline (PBS; Sigma, USA) to lyse the red blood cells. Then the sample were transferred into a 50-mL centrifuge tubes and mixed with 10 mL of 2% BSA in cold PBS to stop the reaction, centrifuged (1,200 rpm, 4°C, 5 min), and washed with cold PBS until it was clear of red blood cells. The remaining white blood cells were collected and resuspended in 1 mL of 50% methanol in deionized water (v/v) on ice for 10 min. During this resuspension, a portion was sampled for total cell count. The cells were then centrifuged (1,500 rpm, 4°C, 5 min), incubated in 1% BSA for 30 min on ice in a 96-well plate at about 10^6^ cells per well, and stained with AlexaFluor488 (A488)-conjugated mouse monoclonal anti-γ-H2AX (lots B19840 and B300815, BioLegend, USA) at a dilution ratio of 1:1.3 in 1% BSA on ice. After 1.5 h, 4 μL of APC-conjugated mouse monoclonal CD45 (lot B250579, BioLegend, USA), which was used to aid the gating of lymphocytes, was added to each well and incubated for a further 30 min on ice. Then nuclear staining was done by incubating the cells in 1:50 dilution of 1 mg/mL Hoechst 33342 (Sigma, USA) in PBS for 5 min on ice. Finally, the cells were washed twice in cold PBS (3,000 rpm, 4°C, 5 min) and stored in 1% paraformaldehyde overnight at 4°C before analysis by flow cytometry the next day. For analysis by confocal microscopy, a portion of each cell suspension was mounted on a glass slide, protected with a cover slip, and sealed with nail polish.

### Analysis of γ-H2AX expression by flow cytometry

White blood cell samples were analyzed on a FACSVerse^TM^ flow cytometer (BD Biosciences) equipped with blue 488 nm and red 635 nm lasers. Instrument performance QC was performed using FACSuite^TM^ CS&T research beads (BD Biosciences). Gating of lymphocyte, granulocyte and monocyte populations was based upon the characteristic populations in the forward vs side scatter as well as the side scatter vs CD45 acquisition plots from analysis of 40,000 white blood cells (S1 Fig). This ensured that a total of about 10,000 lymphocytes were analyzed in each experiment. The A488-labeled γ-H2AX were observed using a 527/32 bandpass filter and CD45PerCP using a 660/10 bandpass filter. Data were acquired and analyzed using FACSuite^TM^ software (Becton Dickinson). Four blind tests were performed on randomly selected four different donors in which blood samples were irradiated with a dose within the range of 0–6 Gy while the analyzing researcher was kept blind to the dose. For intercomparison experiments, portions of the same set of samples randomly selected from six donors were set aside for analysis on FACSCanto II flow cytometer (BD Biosciences) in a participant laboratory by a different operator. Both laboratories in the intercomparison study were general research laboratories; one located in a hospital and the other at a university, that had been operated for at least 10 years. Data collection on both setups were performed by two experienced operators.

### Image acquisition by confocal laser scanning microscopy

Part of the γ-H2AX labeled cells from five donors were set aside from the analysis using flow cytometry for foci counting. Visualization and image acquisition was done on an automated laser scanning confocal microscope (LSM800, Carl Zeiss, Jena, Germany) with a 63X oil immersion objective. Z-stack images covering the whole cells were collected at either 0.3 or 0.7 μm steps. Only cells with a single nucleus and prominent CD45 staining were counted as lymphocytes. A total of 30–50 lymphocytes per dose were collected for foci counting. The diode lasers used in this experiment had wavelengths of 405, 488 and 640 nm for Hoechst 33342 (excitation 343 nm, emission 483 nm), AlexaFluor488 (excitation 494 nm, emission 517 nm) and APC (excitation 650 nm, emission 668 nm), respectively.

### Image analysis of γ-H2AX foci

Foci counting in lymphocytes was performed by manual scoring. Discrete foci were counted as individual entities. Foci that appeared to overlap or merge into a prominent plague were counted as one entity unless the overlapping could be visibly distinguished between the consecutive Z-stacks. Scores were the total foci per cell or the maximum foci per stack for each individual cell.

### Statistical analysis

Data were analyzed by GraphPad Prism version 9.2.0 (GraphPad Software, CA, USA) and SPSS version 18.0 (SPSS Inc, Chicago, IL). Comparison of relative intensities at varying doses was performed using one-way ANOVA and the Tukey’s post hoc test. At the same dose, comparison between two groups was carried out by unpaired student’s *t*-test. Kruskal-Wallis test and the post hoc Dunn’s multiple comparisons test were performed to compare foci counts at varying doses. Linear regression was used for dose estimation. To test the agreement of intensity data from two flow cytometry setups, intraclass correlation coefficient (ICC) was performed. Results were presented as mean (SD) or median (interquartile range). A *p*-value < 0.05 was considered statistically significant.

## Results

### Dose response of lymphocytes and granulocytes by flow cytometry

**Fig 2:**
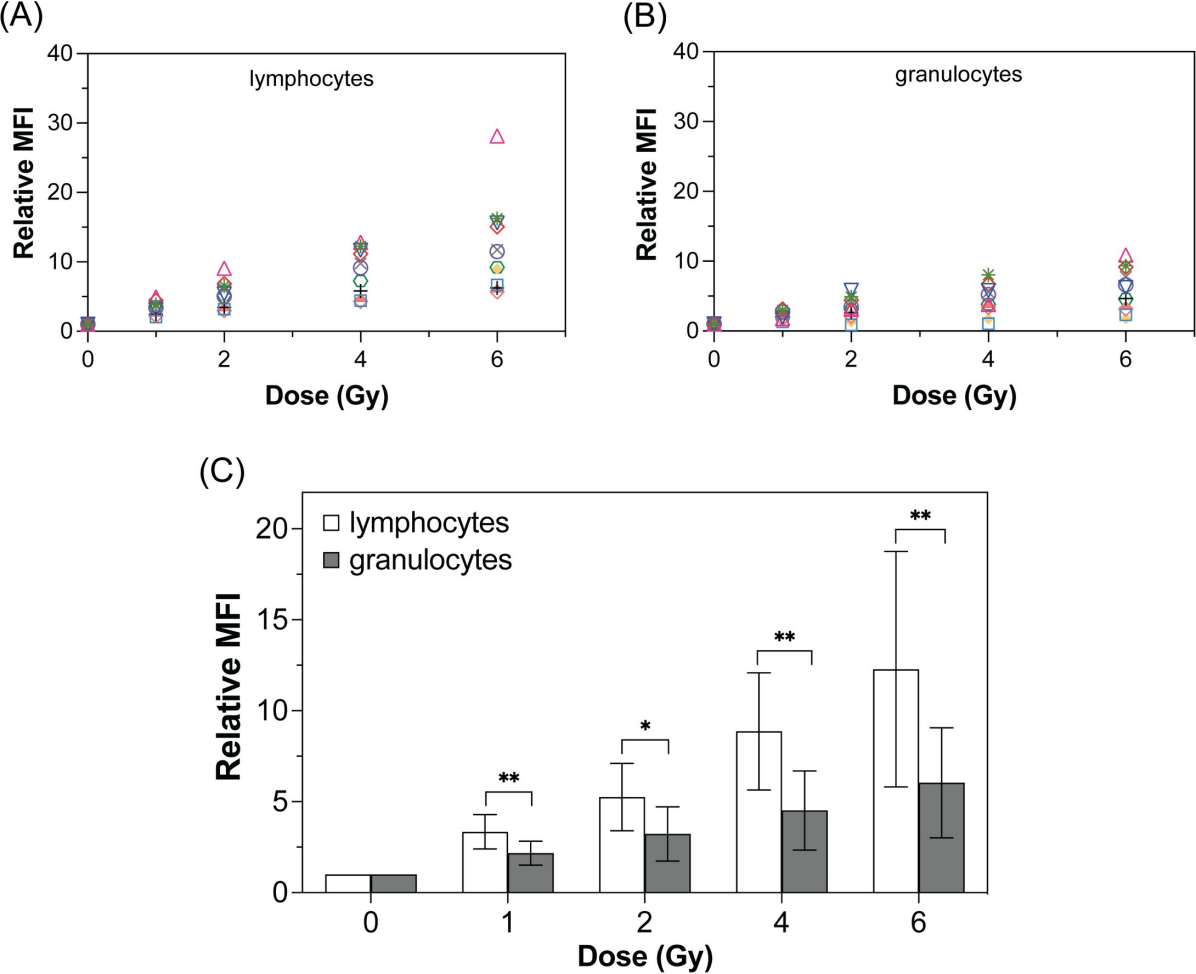
Dose responses of γ-H2AX expression in lymphocytes and granulocytes by flow cytometry. Relative MFI of white blood cell population vs doses for (A) lymphocytes and (B) granulocytes. Each dot in different colors represents an individual donor (n = 11). All *p*-values < 0.05; 0 Gy vs 2, 4, 6 Gy; 1 Gy vs 4, 6 Gy; 2 Gy vs 6 Gy for both lymphocytes and granulocytes. (C) Error bar represents the comparison of relative MFIs of lymphocytes and granulocytes at the same dose. Symbols * and ** correspond to *p-*values of less than 0.05 and 0.01, respectively.

The level of γ-H2AX expression quantitated by the analysis using flow cytometry was represented by the relative mean fluorescence intensity (MFI), which corresponded to the geometric mean fluorescence intensity at irradiation dose normalized by the basal value at 0 Gy. Therefore, the relative MFI data of an individual donor was calculated based on that person’s basal γ-H2AX level in the absence of irradiation. Fig 2 shows the relative MFI of lymphocytes and granulocytes collected from all 11 donors at each irradiation dose. Although the response of γ-H2AX expression to varying doses followed a linear relationship, both sub-populations showed that the relative MFI data points were densely aggregated below an MFI of 5 at 1 Gy, whereas at doses above 2 Gy the data was spread over a much wider range. Comparing the sub-populations at the same dose, the γ-H2AX levels in lymphocytes were significantly higher than granulocytes (*p* < 0.05). Based on this greater γ-H2AX expression in response to absorbed dose, further interlaboratory comparison and foci counting were performed on the gated lymphocyte sub-population.

### Interlaboratory comparison by flow cytometry

**Fig 3:**
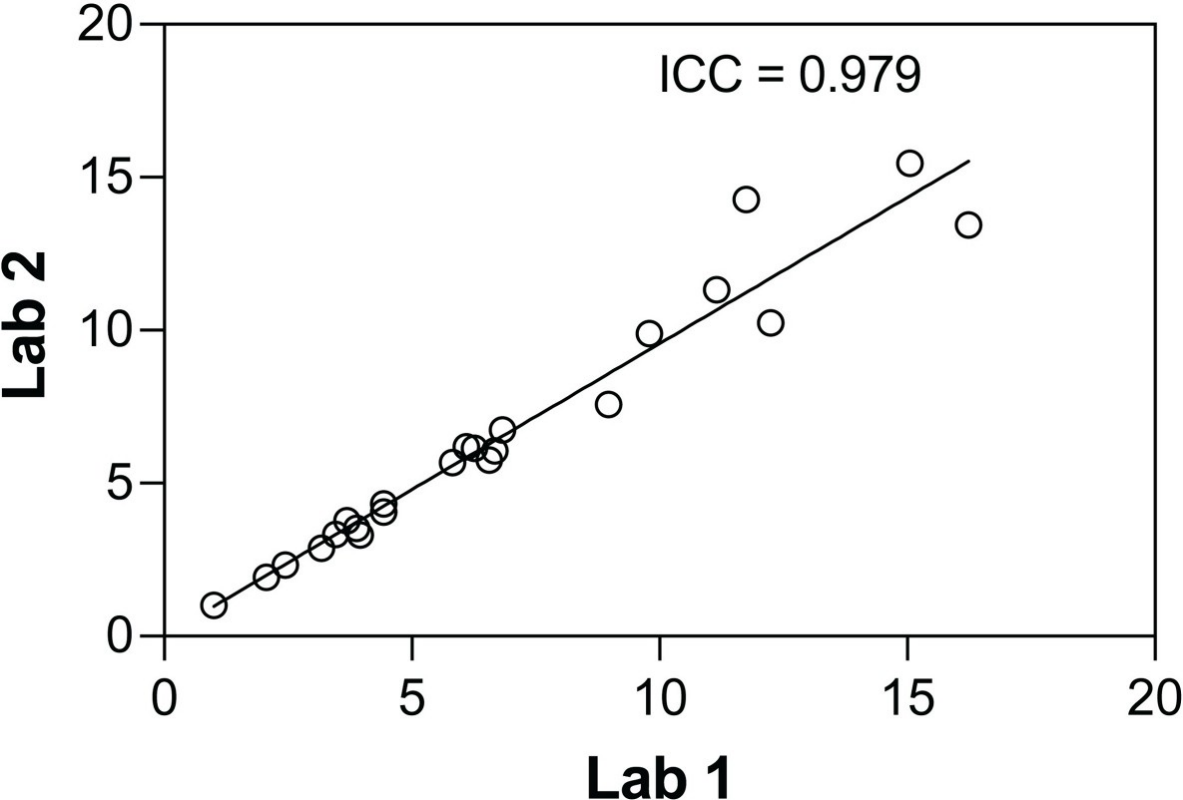
Intraclass correlation of the γ-H2AX expression analyzed by flow cytometry from two different laboratories. The relative MFI results over the dose range from 0 to 6 Gy show the ICC of 0.979 (95% CI, 0.955, 0.990) (n = 6).

To examine the possibility of using different flow cytometry setups in analyzing large scale samples, the interlaboratory comparison study was performed by two different laboratories. The analysis of γ-H2AX expression was conducted on gated lymphocytes from six donors. There was a strong positive correlation between relative MFI results obtained from the two laboratories with ICC of 0.979 (95% CI, 0.955, 0.990) over the dose range from 0 to 6 Gy (Fig 3).

### Average γ-H2AX baseline in the analysis by flow cytometry

**Fig 4:**
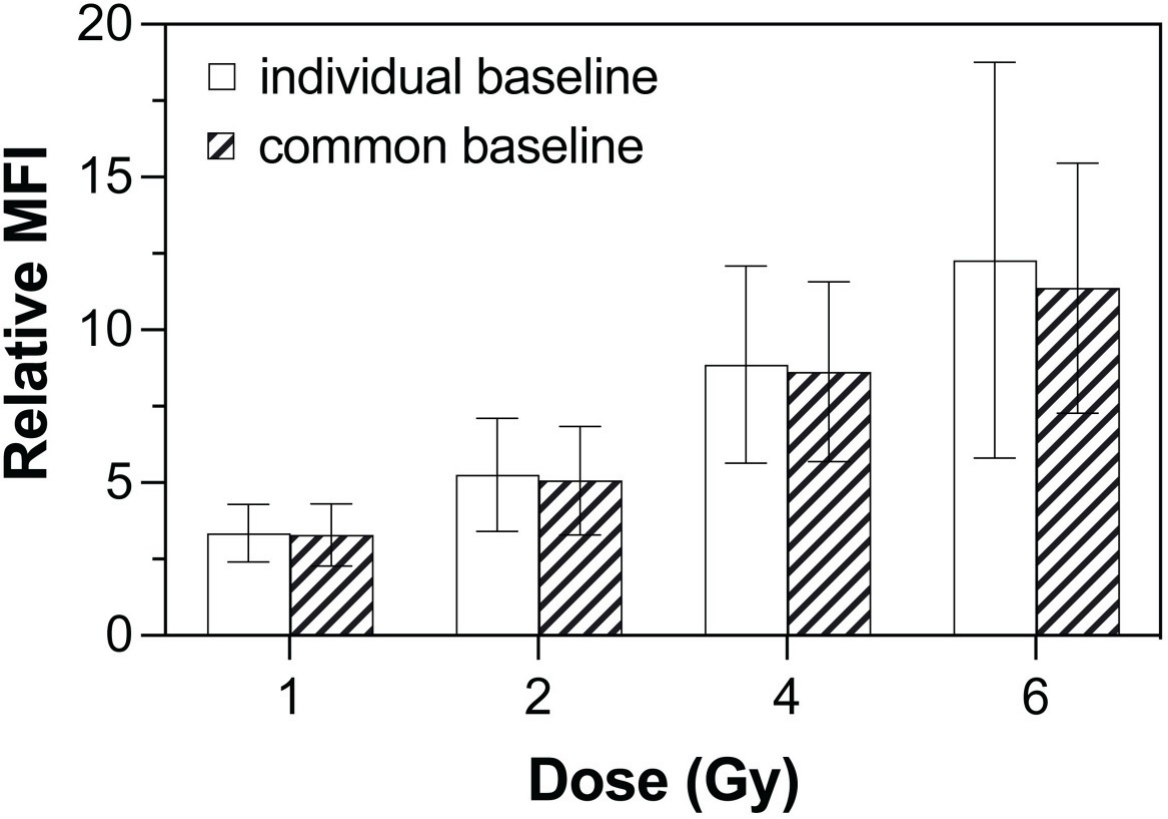
Error bar representing comparison of relative γ-H2AX expression in lymphocytes at each absorbed dose calculated based on individual vs common baseline expression at 0 Gy (n = 11).

To investigate if a common baseline can be used as an alternative to individual baseline in the analysis of γ-H2AX expression by flow cytometry, we compared the relative intensities of gated lymphocytes calculated at each of the absorbed doses from 1 to 6 Gy based on individual vs average MFI of γ-H2AX at 0 Gy (Fig 4). At the same absorbed dose, the relative MFI values obtained by either method were not different for all 11 donors (*p* > 0.05). Therefore, the use of a common basal γ-H2AX value can potentially be applied in the analysis by flow cytometry.

### Formation of γ-H2AX foci and confocal image analysis

**Fig 5:**
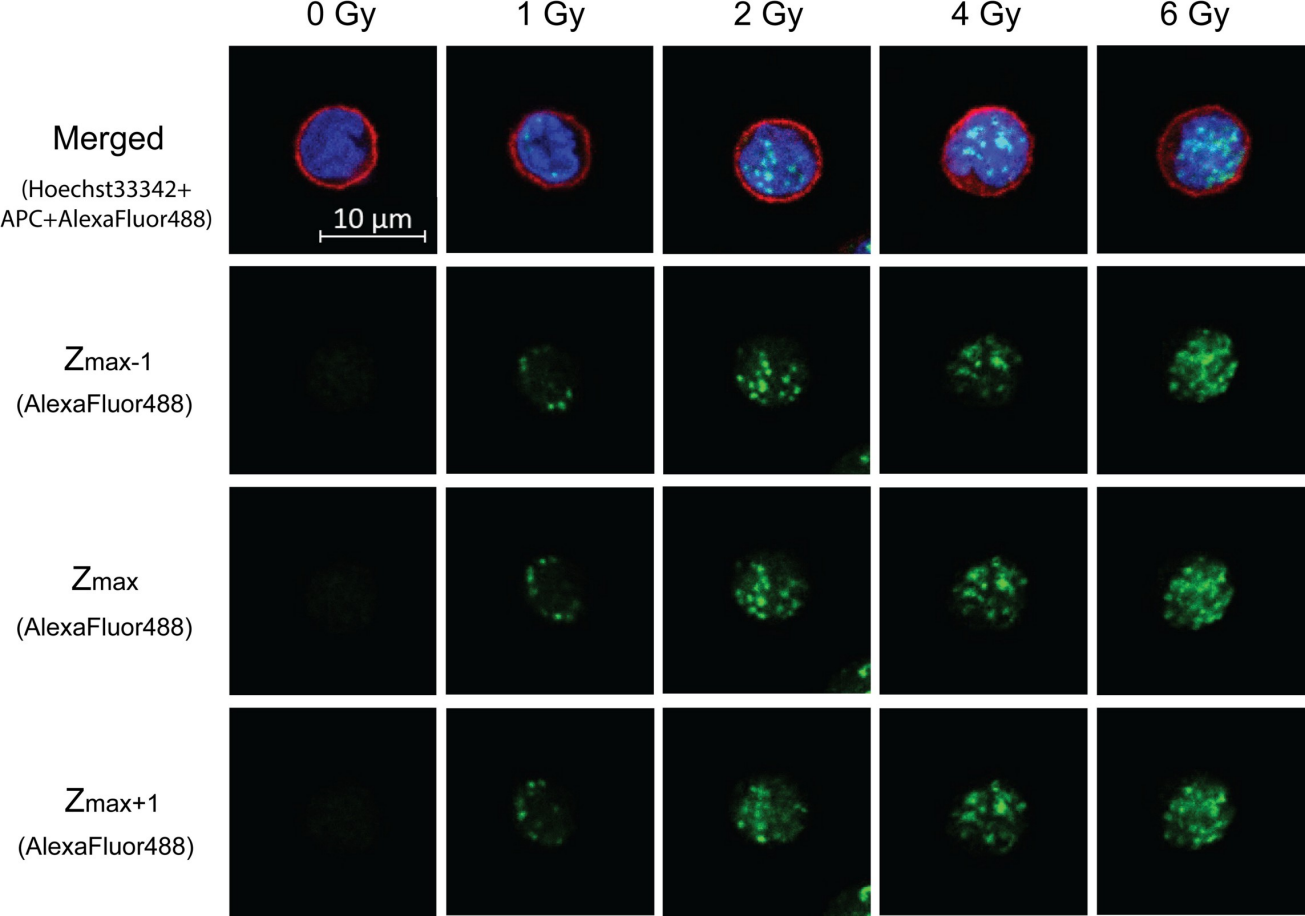
Visualization of γ-H2AX foci after exposure to gamma irradiation by confocal microscopy. Images at each dose were taken at different confocal planes along the cell height at incremental steps of 0.3 μm. In the merged images, γ-H2AX foci are shown in green (AlexaFluor488), nuclei in blue (Hoechst 33342) and cell margins in red (APC). Selected confocal planes Z_max_ are the planes with maximum foci and Z_max-1_ and Z_max+1_ correspond to adjacent confocal planes below and above Z_max_. Scale bar is 10 μm for all images.

Aside from the analysis by flow cytometry, confocal microscopic scoring of γ-H2AX foci was considered as the conventional method to access radiation dose. Analysis using confocal microscopy resulted in higher accuracy compared to fluorescent microscopy [4]. The number of γ-H2AX foci correlates with the degree of DNA damage. An increase in γ-H2AX foci was observed from 1 Gy to 6 Gy (Fig 5). At the lower doses of 1 and 2 Gy, discrete and more frequent foci were observed with 2 Gy compared to 1 Gy. Overlapping foci formation were observed at doses of 4 and 6 Gy, causing difficulty in manual counting of γ-H2AX foci (Fig 5). At each dose, images were taken at different confocal planes throughout the cell height. We found a higher number of foci in the central area of cells due to the larger nuclear cross-sectional area at the center (confocal plane with maximum foci, Z_max_) than near the surface. Fig 5 shows images of Z_max_ and adjacent confocal planes below and above Z_max_ (Z_max-1_, Z_max+1_). Foci counts at each confocal plane along the cell height showed a normal distribution with higher number of foci per planes as well as more planes containing foci observed at higher doses (S2 Fig). Based on this foci distribution, it can be seen that Z_max_ was situated near the middle of cell height.

### Dose response of lymphocytes by confocal microscopy analysis

**Fig 6:**
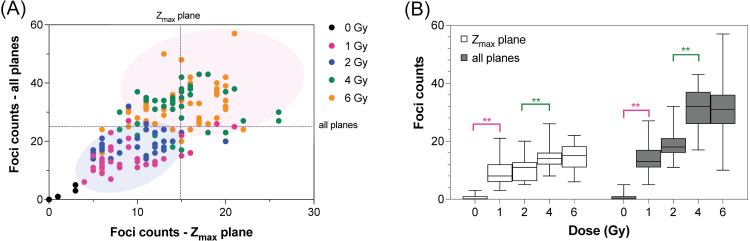
Dose response of lymphocytes using foci counting of microscopy images. (A) Dot plot of number of foci counts from all planes (y-axis) and Z_max_ plane (x-axis) (n = 5, 30–50 lymphocytes per dose). Blue and pink shadows highlight the two distinct regions where data are aggregated. (B) Box plot of foci counts as a function of dose. All *p*-values < 0.01; 0 Gy vs 1, 2, 4, 6 Gy; 1 Gy vs 4, 6 Gy; 2 Gy vs 4, 6 Gy for both all planes and Z_max_ plane. Asterisks (**) refer to *p* < 0.01 for comparison between adjacent doses (0 vs 1 Gy and 2 vs 4 Gy).

Quantitation of γ-H2AX foci was performed by manual counting of confocal images in which each discrete focus on a confocal plane along the cell height was counted as ‘1’, and the resurfacing focus from a previous adjacent plane onto the plane of counting was excluded. Fig 6A shows a dot plot of total number of foci per cell and number of foci per plane taken from Z_max_ at each dose. A correlation between different representations of foci count was clearly observed. Therefore, the number of foci per plane taken from Z_max_ could be used as a representative of radiation-induced γ-H2AX expression at that absorbed dose. Interestingly, data clustered into two distinct regions: 1–2 Gy (Fig 6A, blue and pink dots, blue shadow) and 4–6 Gy (Fig 6A, green and yellow dots, pink shadow). Of these two sets, data in the 1–2 Gy region were more aggregated, while those in the 4–6 Gy spread over a larger area, which might reflect the inter-individual variation with dose observed in the analysis using flow cytometry.

To set the boundaries between the two regions, lines were made at 25 foci/cell on the y-axis and 15 foci/plane on the x-axis, respectively, to include > 90% of foci counts at 1 and 2 Gy within the 1–2 Gy region. Based on the boundary associated with foci per plane, some of the foci counts from the 4–6 Gy region fell below the set limit and into the 1–2 Gy region. By contrast, the boundary associated with total foci per cell better excluded such penetration of values from higher doses.

Fig 6B shows as box plot with the same data as in the dot plot, but as a function of dose. The proportional increase in foci counts with doses was observed from 0 to 4 Gy before leveling off at 6 Gy for both total foci per cell (Fig 6B, gray bars) and foci per plane (Fig 6B, white bars). In addition, the Kruskal-Wallis test with a post-hoc Dunn’s for multiple comparison also showed that both methods of foci scoring could differentiate doses between adjacent doses: 0 vs 1 Gy (all planes: *p* = 0.0025, Z_max_ plane: *p* = 0.0001) and 2 vs 4 Gy (all planes: *p* = 0.0001, Z_max_ plane: *p* = 0.0047).

### Testing of γ-H2AX assay as a dose estimation by flow cytometry

**Fig 7:**
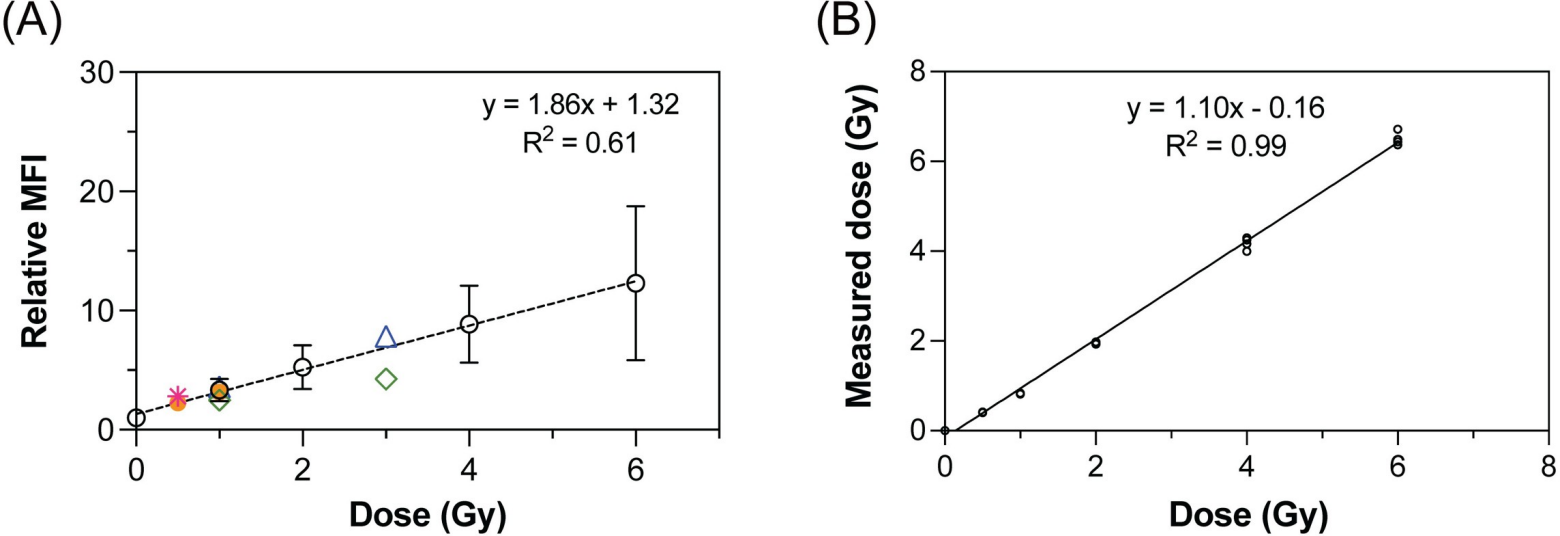
Blind testing of dose response curve. (A) flow cytometry and (B) physical dosimeter (nanoDots^TM^). Colored symbols in open triangle, open diamond, closed circle and asterisk represent data obtained from the blind tests conducted on different dates. The same symbol at different selected doses in (A) corresponds to the same donor.

Blind tests were conducted to test the accuracy of the dose response curve. Linear regression was performed on the relative intensity data obtained from all 11 donors on the dose response curve (Fig 7A). Three doses, 0.5, 1 and 3 Gy, were selected to cover the range of low, moderate, and high levels of absorbed dose in blind tests. The estimated doses were shown in colored data points and deviation from their corresponding actual values would be referred to as the mean absolute difference. At doses ≤ 1 Gy, the estimated doses of 0.50 and 0.81 Gy were obtained for the actual dose of 0.5 Gy, and the estimated doses of 0.62, 0.98 and 1.23 Gy were obtained for the actual dose of 1 Gy. At the 3 Gy dose, the estimated doses were 1.60 and 3.51 Gy. Based on these results, only the estimated doses obtained from the actual dose of 3 Gy exhibited the mean absolute differences of more than 0.5 Gy. As a comparison, the measurement of absorbed doses by a physical dosimeter strongly correlated (R^2^ = 0.997) to the actual values over the range of 0 to 6 Gy (Fig 7B). Nonetheless, it can still be seen that the dose response curve based on the relative intensities provides a relatively good estimation within 0.5 Gy difference from the actual value at doses ≤ 1 Gy in the absence of a physical dosimeter.

## Discussion

In the present study, we examined the use of γ-H2AX as a quick tool to screen for individuals with possible acute radiation exposure. Previous studies have shown that the threshold level of exposure that can lead to the onset of radiation sickness is about 0.75 to 1 Gy [3], whereas greater biological damage, higher risk of acute radiation, and less than 90% probability of survival without therapy occur at doses above 2 Gy [36]. Therefore, the ability to quickly determine and assign individuals to appropriate medical treatments can save their lives. In the case of a radiation emergency, this requires an integrated ensemble of biological assays for triage rather than a stand-alone one [3]. Due to its high sensitivity to IR, small sample volume requirement with minimal invasiveness, quick sample preparation, and high-throughput capacity, γ-H2AX has emerged as a robust biomarker and remains an ongoing research interest in biodosimetry [4,32].

As the γ-H2AX expression typically increases after 30 min and peaks at 2 h after exposure to IR before decaying due to repair of DSBs [20,22,37], the 45 min timeframe between radiation and processing of whole blood in our experiments was well within this peak window. Although our work showed a detectable γ-H2AX response in granulocytes by flow cytometry and also microscopy (S3 Fig), it was at a lower level compared to lymphocytes. This can be due to the lack of DNA-dependent protein kinase that is responsible for the generation of γ-H2AX in polymorphonuclear leukocytes [18,38]. Given the lower level of response and the notion of impaired DNA damage signaling associated with granulocytes, we will focus our interpretation and discussion of data in our study only on the lymphocytes. In terms of the dose response curve generated by flow cytometry, a linear trend of increasing relative intensities with increasing absorbed doses shown in our study is similar to previous reports [1,12,16,19]. Based on blind tests, our dose response curve may be applied for a quick determination of exposure doses below 1 Gy with an uncertainty within 0.5 Gy that is regarded as a precision for making clinical decision in triage [8]. However, the large inter-individual variation observed at doses above 2 Gy, may be attributed to the difference in individual radiosensitivity that becomes more apparent with increasing DNA damage [19].

Because relative γ-H2AX intensities are used to represent the cellular responses to DNA damage caused by IR, the use of relative MFI for the quantitation of γ-H2AX levels in individuals can be limited due to the impracticality of performing multiple blood draws. Similarly, in the lymphocyte depletion kinetic (LDK) assay that is proposed as the early method in the combined biological assays for medical triage [3], the knowledge of individual’s baseline on lymphocyte count is also needed to estimate the absorbed dose. Our results (see Fig 4) demonstrated that relative intensities were not statistically different whether individual or average γ-H2AX expression at 0 Gy was used as a reference. Therefore, a common baseline may potentially be used in the analysis using flow cytometry in case of a radiation emergency when the knowledge of individual’s baseline is unavailable.

The only two laboratories participated in the intercomparison study presents the limitation of our work. Compared to the previous intercomparison studies that used the foci scoring method [39–42], at least four laboratories participated in the studies in which a standard protocol on processing and staining of samples as well as criteria on foci scoring were provided for each laboratory. As our analysis of γ-H2AX intensities by flow cytometry was carried out by only two laboratories in this present study, we cannot yet generalize the outcome of our work. More participating laboratories are required in future work to validate the use of this technique in handling triage. Moreover, the availability of antibody, fluorophores, and the experience of scorer can also be accounted for the limitation of fluorescence-based techniques in general [41], and these should be taken into consideration when perform the intercomparison study besides the use of standardized protocol on processing and staining [40].

Despite the laborious nature of foci scoring from microscopy images, the analysis provides data that are characteristic of cells [12] in which the extent of DNA DSBs can be examined by both the morphology and number of foci. Because the manual counting of foci was performed on 30–50 lymphocytes per dose, this presents the weakness of our work compared to previous studies that counted more cells [40,41,43]. Nevertheless, the results of foci counts from all confocal planes in Fig 6B still agreed with those previous reports that observed a linear dose response of foci number increasing approximately 10–15 foci/cell per Gy in the dose range of 0–2 Gy within 1 h post exposure [1,12,21,43,44]. In addition, as we demonstrated in our study that scoring from the Z_max_ plane was correlated with scoring from all planes, Z_max_ plane could be used as an alternative for dose estimation. Thus, scoring of foci from Z_max_ may imply the use of epifluorescence microscopy that is more common in many laboratories. At the highest dose used in this study, the saturation of foci counts could be attributed to the merging of neighboring, discrete foci into larger plagues (see Fig 5) similar to the observation of overlapping foci especially at doses above 2 Gy reported in a previous study that quantified foci counts at 1 h post irradiation [1]. This in turn might have reflected the extent of DNA DSBs to size rather than number of foci at high doses. Based on these results, scoring from the Z_max_ plane may be applied as a quick determination of absorbed doses below or above 2 Gy (see Fig 6B, white bars 2 vs 4 Gy) that correspond to the level of moderate exposure [36] in the case of a radiation emergency when time is of concern in triage.

Due to the kinetic disappearance of the γ-H2AX signal as DNA repair progresses, the γ-H2AX assay has to be implemented quickly post-IR exposure. After DNA DSBs, the γ-H2AX signal rises within 30 min to its peak at ~ 1–2 h [22] before sharply decaying with an approximated half-life of 1.5–1.9 h [1,12,13]. Both cytometry and microscopy analyses published in previous work [12,18,20,22] show that γ-H2AX expression returns to near baseline within 4–7 h of IR exposure. However, at 24 h post-IR exposure, the γ-H2AX signal after a 4 Gy dose is still detected by flow cytometry [18] and after a 2 Gy dose by foci counting [12]. Simulation by Horn *et al*. [13] confirmed that microscopy is more sensitive than cytometry for detection of minimum absorbed doses up to 96 h post-IR exposure. Therefore, flow cytometry can be useful if the samples are collected within the first few hours (< 4 h) after IR exposure, and more practical for occupation-related radiological incidents that involve tens to hundreds of people. At later time points, but still within a few days of IR exposure, microscopy is more appropriate for quantitation of residual foci that are hard to repair. Several studies [1,13,32] have developed mathematical methods that include kinetics of foci formation and decay in the estimation of absorbed doses when calibration curves are not available. However, parameters such as background foci number, residual foci number and repair kinetics must be determined prior to usage [1,32].

Although much work has been done on the investigation of γ-H2AX dose response curves [6], a consensus on the implementation of the assay has not been reached. Important questions remain: how many calibration curves at what time points do we need to make, what dose limit can cytometry and microscopy distinguish during those time periods, and how best to establish a workflow that integrates γ-H2AX with other biological assays. In terms of radiation emergencies, the earliest access to samples may be within 24 h. As experiments were only conducted during the peak window of γ-H2AX expression, it is of interest to test both cytometry and microscopy approaches at other time points post-IR exposure, for example at 2, 8 and 24 h. Then, at time points after 24 h but within the first few days we can study foci counting and quantitative modeling.

## Conclusion

Analysis of γ-H2AX expression by both flow cytometry and foci counting of confocal microscopic images at early timepoints can be used as a quick dose estimation of radiation exposure with the capability to differentiate the absorbed dose. Pre-determined values of endogenous γ-H2AX expression may be applied in flow cytometry analysis. While flow cytometry provided sensitivity for dose estimations at lower doses up to 1 Gy, the foci number and morphology appeared useful in reassuring the estimation of doses above 2 Gy when inter-individual variation affected the cytometry analysis. This rapid estimation of absorbed dose can inform radiation triage and help in prioritizing medical treatments to exposed individuals. In summary, the γ-H2AX assay presented a reliable and promising technique to quickly assess biological responses to IR at early timepoints. The establishment of methods and dose-response curves from various biological assays, as well as an organized workflow within the national biodosimetry network, is needed for radiation emergency preparedness.

## Supporting information

S1 Fig Representatives of the gating on lymphocytes and granulocytes based on side scatter and CD45 and the corresponding γ-H2AX histograms analyzed by flow cytometry. (TIF)

S2 Fig Representatives of foci distribution over different focal planes at doses 1, 2 and 6 Gy. Note: Each bar refers to the number of foci per plane. Total foci per cell refers to the cumulative number of foci excluding those that resurface across consecutive Z-stacks. Z_max_ plane refers to the plane with maximum number of foci.
(TIF)

S3 Fig Confocal images of polymorphonuclear cells at all doses from 0–6 Gy. (TIF)

S1 Table Blood samples used in the experiments. Note: In the blind test column, 0.5, 1 and 3 refers to the doses in Gy being tested.
(TIF)
