# Supplementary figures and images for: Quantification of histone H2AX phosphorylation in white blood cells induced by ex vivo gamma irradiation of whole blood by both flow cytometry and foci counting as a dose estimation in rapid triage
Source: PLoS One. 2022 Mar 23;17(3):e0265643. doi: 10.1371/journal.pone.0265643 (PMC8942256; doi:10.1371/journal.pone.0265643)

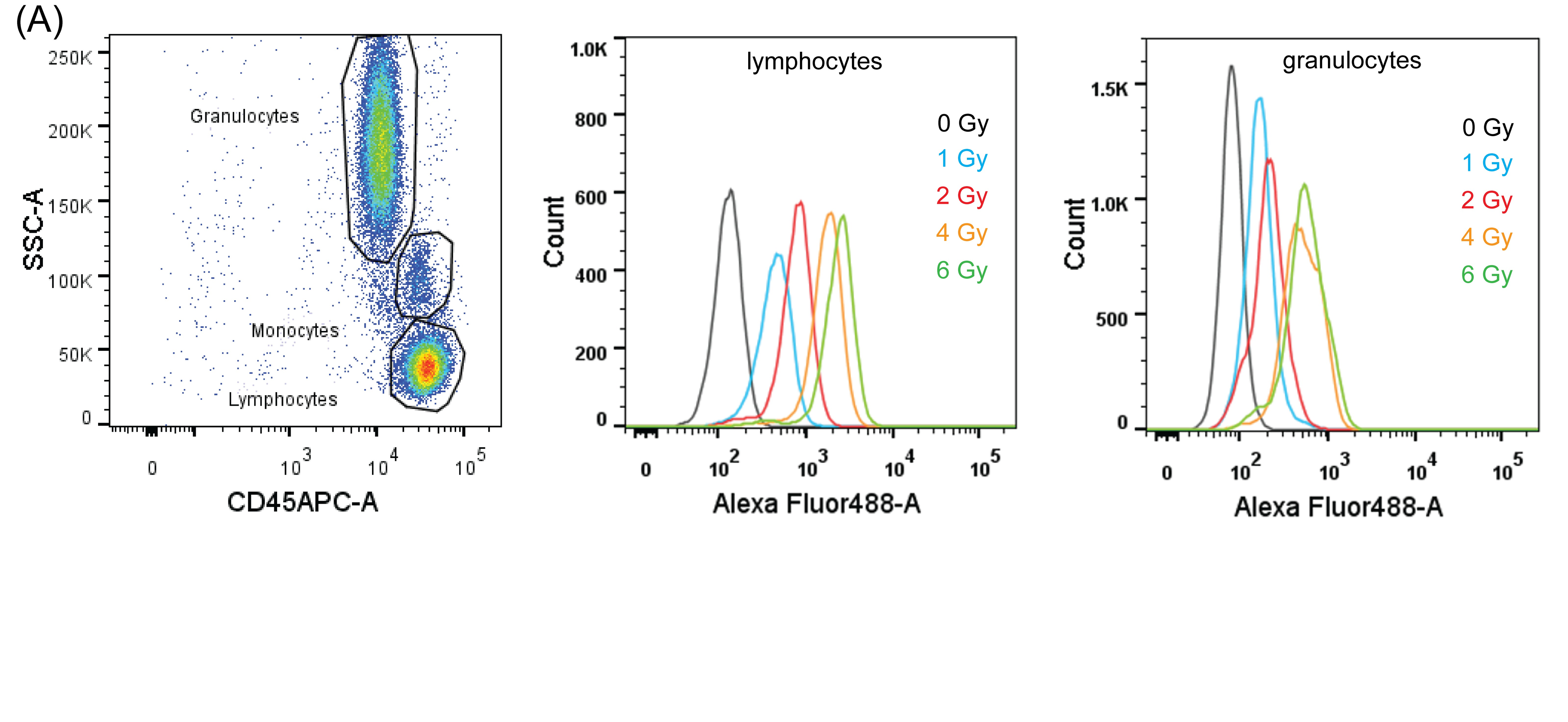

Supplement: S1 Fig — (TIF) [file pone.0265643.s001.tif]

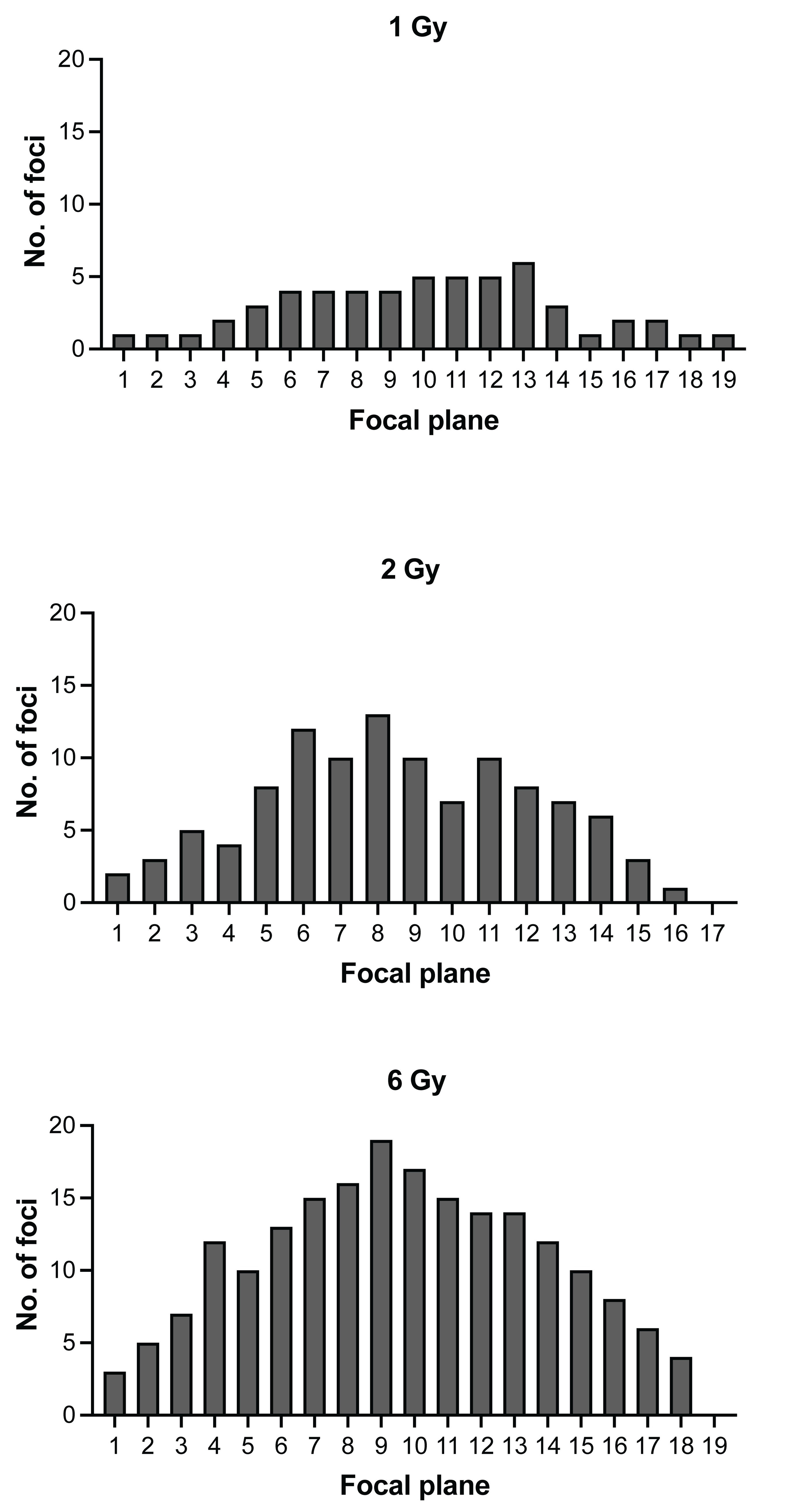

Supplement: S2 Fig — Note: Each bar refers to the number of foci per plane. Total foci per cell refers to the cumulative number of foci excluding those that resurface across consecutive Z-stacks. Zmax plane refers to the plane with maximum number of foci. (TIF) [file pone.0265643.s002.tif]

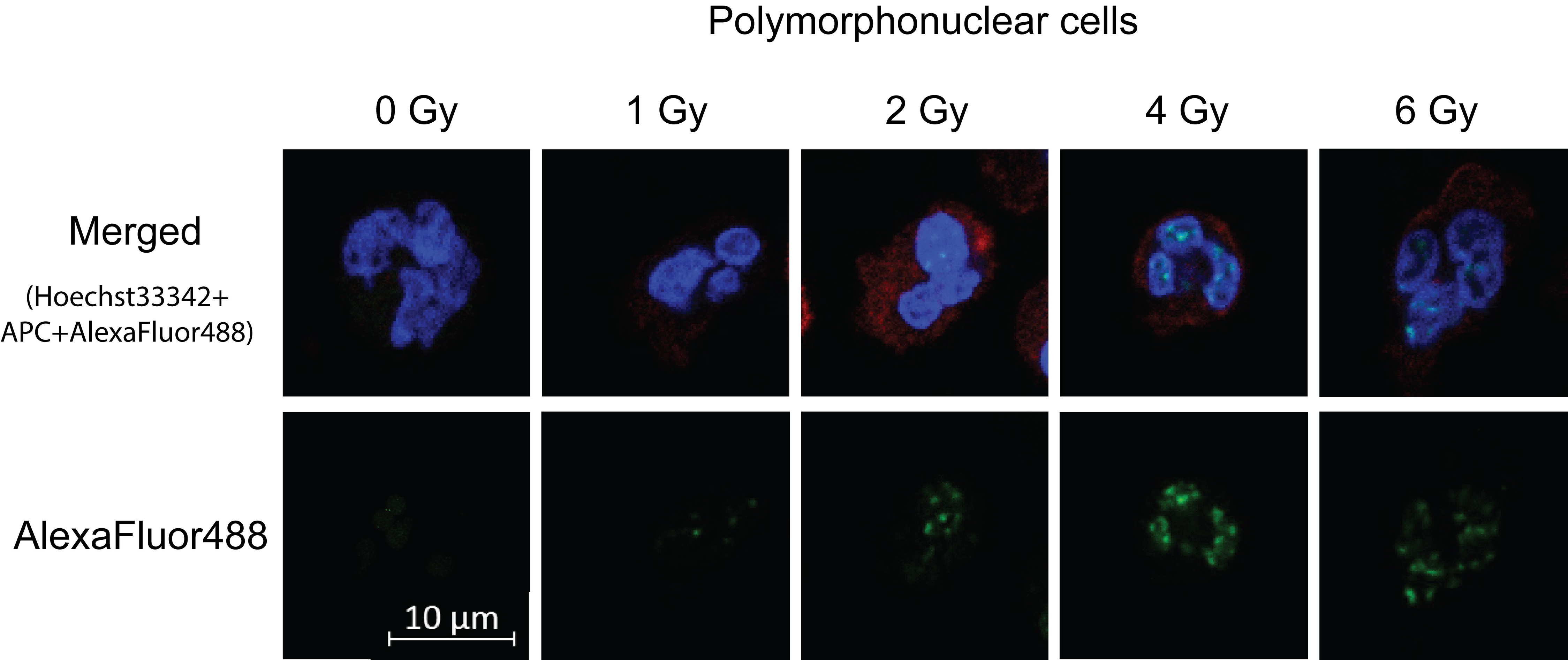

Supplement: S3 Fig — (TIF) [file pone.0265643.s003.tif]

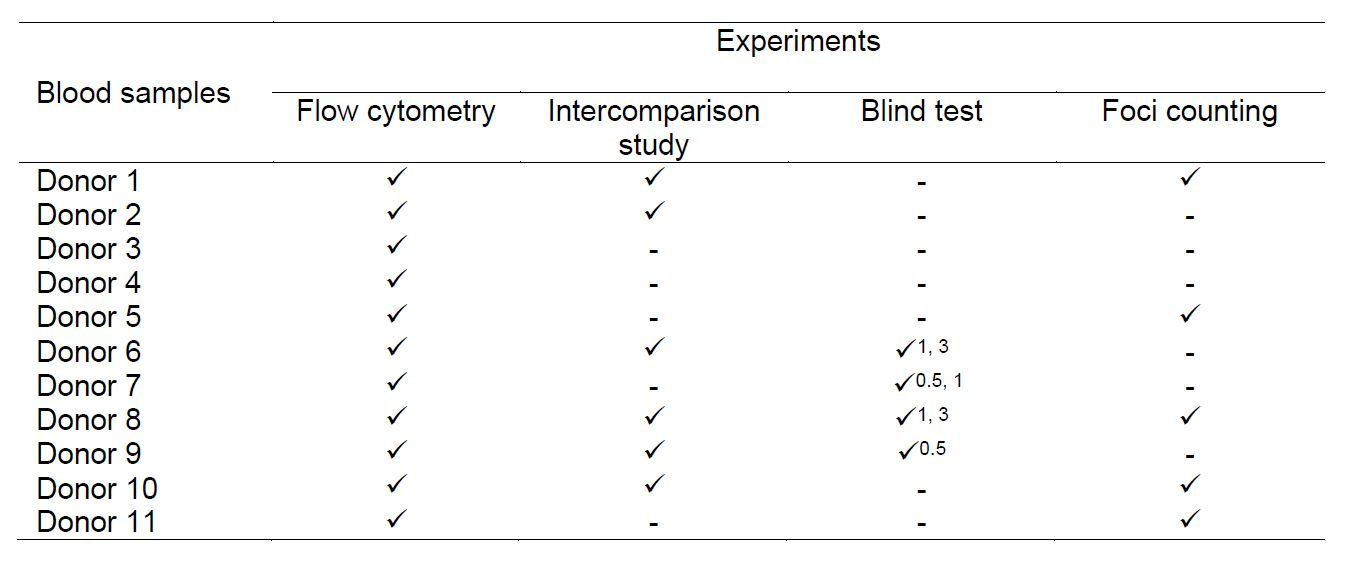

Supplement: S1 Table — Note: In the blind test column, 0.5, 1 and 3 refers to the doses in Gy being tested. (TIF) [file pone.0265643.s004.tif]
